# Comparative Evaluation of Methods for Estimating Retinal Ganglion Cell Loss in Retinal Sections and Wholemounts

**DOI:** 10.1371/journal.pone.0110612

**Published:** 2014-10-24

**Authors:** Ben Mead, Adam Thompson, Ben A. Scheven, Ann Logan, Martin Berry, Wendy Leadbeater

**Affiliations:** 1 Neurotrauma Research Group, Neurobiology Section, School of Clinical and Experimental Medicine, University of Birmingham, Birmingham, United Kingdom; 2 School of Dentistry, University of Birmingham, Birmingham, United Kingdom; NIH/NEI, United States of America

## Abstract

To investigate the reliability of different methods of quantifying retinal ganglion cells (RGCs) in rat retinal sections and wholemounts from eyes with either intact optic nerves or those axotomised after optic nerve crush (ONC). Adult rats received a unilateral ONC and after 21 days the numbers of Brn3a^+^, βIII-tubulin^+^ and Islet-1^+^ RGCs were quantified in either retinal radial sections or wholemounts in which FluoroGold (FG) was injected 48 h before harvesting. Phenotypic antibody markers were used to distinguish RGCs from astrocytes, macrophages/microglia and amacrine cells. In wholemounted retinae, counts of FG^+^ and Brn3a^+^ RGCs were of similar magnitude in eyes with intact optic nerves and were similarly reduced after ONC. Larger differences in RGC number were detected between intact and ONC groups when images were taken closer to the optic nerve head. In radial sections, Brn3a did not stain astrocytes, macrophages/microglia or amacrine cells, whereas βIII-tubulin and Islet-1 did localize to amacrine cells as well as RGCs. The numbers of βIII-tubulin^+^ RGCs was greater than Brn3a^+^ RGCs, both in retinae from eyes with intact optic nerves and eyes 21 days after ONC. Islet-1 staining also overestimated the number of RGCs compared to Brn3a, but only after ONC. Estimates of RGC loss were similar in Brn3a-stained radial retinal sections compared to both Brn3a-stained wholemounts and retinal wholemounts in which RGCs were backfilled with FG, with sections having the added advantage of reducing experimental animal usage.

## Introduction

Retinal ganglion cells (RGCs) populate the ganglion cell layer (GCL) of the retina, although in some species a few are found in the inner nuclear layer (INL; displaced RGCs) [1]. All rat RGC axons become myelinated at the lamina cribrosa [2], [3], project centripetally in the optic nerve, partially decussate in the chiasma and synapse in the lateral geniculate body, superior colliculus, hypothalamus and pretectal area [3]; a few non-/thinly-myelinated peptidergic axons course centrifugally in the optic nerve [4]. Accurate estimates of the total number of RGCs are compromised by counting errors engendered by: (1), the presence of astrocytes and displaced amacrine cells, estimated to contribute up to 50% of cells in the GCL [5], [6] and which co-localize with many markers used to identify RGCs [7]–[9]; (2), RGCs displaced into the INL [1]; and (3), a progressive decrement in RGC density from the centre of the retina to the periphery [10].

Techniques aimed at overcoming these confounding issues include: (1), use of optimal detection and sampling methods; (2), counting myelinated RGC axons in the optic nerve; (3), using phenotypic antibody markers with exclusive affinity for RGCs; (4), transfecting RGCs with green fluorescent protein under control of the Thy-1 promoter; and (5), back filling exclusively RGCs with retrogradely transported axon tracers such as FluoroGold (FG) injected into an optic nerve or both superior colliculi, for which 98% of RGC project to [3], [11]–[13]. The precision of estimates of total RGC numbers from either retinal sections or wholemounts relies on the sampling method used. In wholemounts, the location of standard sampling areas along superior, inferior, temporal and nasal radii is designed to account for declining RGC/mm^2^ at increasing radial distances. In retinal sections, sampling of RGC/mm over a standard length of the GCL in matching sections between animals is required and consistency is best achieved by counting RGCs in radial retinal sections through the optic nerve head. For wholemounts, total absolute counts of RGC number is possible [10] yet may be erroneous if calculated from samples in which the spatial differences in RGC density are not taken into account when correcting for total retinal area, or if the retina is not dissected accurately. Nonetheless, in RGC viability studies it is generally accepted that retinal wholemount and sectional quantitative data give acceptable estimates of percentage differences in RGC numbers between treatment and control groups.

Myelinated axon counts from the optic nerve yield good estimates of the total number of RGCs in the retina [14], but monoaminergic small diameter efferent axons with thin myelin sheaths [4] are difficult to differentiate from the axons of small RGCs and thus total myelinated axon counts overestimate RGC frequency. The use of antibody phenotypic markers to identify RGCs, such as βIII-tubulin [15], [16], Islet-1 [7], Thy-1 [17] and Brn3a [18] is critically dependant on antibody specificity. Thy-1 is down-regulated in RGCs in the diseased retina [19] where this antibody underestimates RGC number and βIII-tubulin and Islet-1 antibodies both cross-react with ligands expressed by amacrine cells [7]–[9], [15] which are less likely to die than RGCs after ONC [20], and thus both antibodies exaggerate RGC survival in cases of e.g. glaucoma and optic nerve neuritis. By contrast, Brn3a exclusively stains RGCs by binding to a RGC specific nuclear epitope [18] with some exceptions such as the intrinsically photosensitive RGC that are found to be Brn3a^-^ [21]. Transfection of RGCs with the green fluorescent protein gene (*gfp*), using the Thy-1 promoter is a function of transfection efficiency and the number of cells labelled is rarely absolute, e.g. intravitreal injected adeno-assosciated virus (AAV) vectors transfect <85% RGCs [22].

Back filling all RGCs with FG requires either bilateral injection into the superior colliculi to capture both contralateral and ipsilateral RGC projections [11], [23], or unilateral injection into the optic nerve [3], [24]. The technique has the potential drawbacks of: (1), gap junctional transfer of tracer between coupled cells in the GCL [12] (although transfer takes significantly longer into amacrine cells than into RGCs); (2), incomplete uptake of tracer by RGC axons at the site of injection; (3), possible toxicity [25]; (4), erroneous counting of tracer-filled macrophages/microglia which have phagocytised dying/dead FG^+^ RGC [26]; and (5), the lack of persistence of FG in neuronal cytoplasm [27], including RGCs 3 weeks after administration to the superior colliculus [11]. Since 2.5–4.2% of albino/hooded rat RGC axons project ipsilaterally to the superior colliculus [21], respectively, FG retrograde transport after injection of one superior colliculus underestimates RGC numbers in the contralateral retina in this species [28].

Another disadvantage of labelling RGCs with FG in retinal whole mount analysis is that the entire retina and optic nerve are used to generate the labelled cells leaving no tissue for extending the analysis to include correlative axon counts in the optic nerve and wider cellular and molecular evaluations of treatments, making retinal section analysis more efficient both in terms of data acquisition and animal usage [7], [9], [24]. However, it is not clear how reliable counting RGCs in retinal sections is compared to wholemounts where a much larger sample of the retina is analysed.

The present study aimed to define and compare the reliability of RGC counting methods including FG RGC back-filling and immuno-staining with the commonly used phenotypic markers βIII-tubulin, Islet-1 and Brn3a in rat retinal wholemounts and radial sections to determine the most reliable estimates of RGC loss after induction of RGC death by ONC.

## Materials and Methods

All reagents were purchased from Sigma (Poole, UK) unless otherwise specified.

### Experimental design

The left optic nerve in a total of 12 rats was crushed (ONC), the right optic nerve remained intact (controls). Animals were separated into 2 groups (Table 1), each of 6 rats, which were all euthanized on day 21 after ONC. In Group 1, FG was injected into the proximal segment of both optic nerves of each rat at day 19 and, 48 h later, FG back-filled RGCs, along with Brn3a-stained RGCs were counted in retinal whole mounts. Group 1 comprised retinae from right eyes with an intact optic nerve (Group 1a) and retinae from left eyes that received an ONC (Group 1b). In Group 2, βIII-tubulin-, Islet-1- and Brn3a-stained RGCs were counted in radial sections of the retinae through the optic disk and double stained for astrocytes using glial fibrillary acidic protein (GFAP) amacrine cells using syntaxin-1 [29], [30], and macrophages/microglia using ED1 (Table 2). Group 2 comprised retinae from right eyes with an intact optic nerve (Group 2a) and retinae from left eyes that received an ONC (Group 2b).

**Table 1:** Animal grouping.

Group 1 (6 rats)	RGC counted in wholemounted retinae	Intact optic nerve	Group 1a (6 eyes)
		Optic nerve crush	Group 1b (6 eyes)
Group 2 (6 rats)	RGC counted in radial retinal sections	Intact optic nerve	Group 2a (6 eyes)
		Optic nerve crush	Group 2b (6 eyes)

**Table 2:** Antibodies used in immunohistochemistry.

Antigen	Specificity	Host species	Dilution	Supplier	Catalogue no.
βIII-tubulin (sections)	Monoclonal	Mouse	1∶500	Sigma	#T8660
Brn3a (sections)	Polyclonal	Goat	1∶200	Santa Cruz (Santa Cruz, CA)	#SC-31984
Islet-1 (sections & wholemounts)	Polyclonal	Rabbit	1∶200	Abcam (Cambridge, UK)	#ab20670
Brn3a (wholemounts)	Polyclonal	Goat	1∶100	Santa Cruz (Santa Cruz, CA)	#SC-31984
Syntaxin-1	Monoclonal	Mouse	1∶200	Abcam	#ab3265
GFAP	Monoclonal	Mouse	1∶200	Sigma	#G3893
ED1	Monoclonal	Mouse	1∶200	Serotec (Oxford, UK)	#MCA341R
Mouse IgG (Fluor 488)	Polyclonal	Donkey	1∶400	Molecular probes (Paisley, UK)	#A-21202
Rabbit IgG (Fluor 488)	Polyclonal	Donkey	1∶400	Molecular Probes	#A-21206
Rabbit IgG (Fluor 594)	Polyclonal	Donkey	1∶400	Molecular Probes	#A-21207
Goat IgG (Fluor 594)	Polyclonal	Donkey	1∶400	Molecular Probes	#A-11058

### Animals

All animal procedures were performed in strict accordance to the UK Home Office Animals Scientific Procedures Act, 1986 and approved by the University of Birmingham Ethical Review Sub-Committee. Twelve adult female Sprague Dawley rats weighing 150–200 g (8–10 weeks; Charles River, Kent, UK) were housed in conditions of 21°C and 55% humidity under a 12 h light and dark cycle, given food/water *ad libitum* and supervised constantly by trained staff. Anaesthesia was induced with 5% Isoflurane/1.5l per min O_2_ (National Veterinary Supplies, Stoke, UK) and maintained at 3.5% during surgery.

### Surgical procedures

After anaesthetic induction as described above and a subcutaneous injection of buprenorphine (0.1 ml/100 g; National Veterinary Supplies) animals were secured in a head-holding frame. Intraorbital left ONC was performed in Group 1b and 2b 8–10 week old rats as described previously [31]. Briefly, the optic nerve was exposed and crushed using forceps 1 mm posterior to the lamina cribrosa, completely closed around the optic nerve for 5 seconds, without damaging the central retinal artery (confirmed by lack of ischemia in eyes 7–21 days after ONC). After surgery, animals were placed in warmed (30°C) recovery cages and closely monitored until the return of normal behaviour, when they were transferred to home cages. Two days before tissue harvest, all Group 1 animals were re-anaesthetised and the optic nerves re-exposed as above and 2 µl of 4% FG solution (Biotium, Hayward, CA) in sterile phosphate-buffered saline (PBS) was injected directly into the right and left nerves distal to the lamina cribrosa (proximal to the crush site in the left optic nerves in Group 1b), using a glass micropipette, produced in-house from a glass capillary rod (Harvard Apparatus, Kent, UK) using a Flaming-Brown micropipette puller (Sutter Instruments, Novato, CA). The injected FG is incorporated into axons and retrogradely transported axonally to RGC somata.

### Tissue preparation

Group 1 rats were euthanized at 21 days by rising concentration of CO_2_. After removal of the cornea and lens, the residual eye cups were immersion fixed in 4% paraformaldehyde (PFA; TAAB, Reading, UK) in PBS for 2 h at 4°C before the retinae were removed and flattened onto Superfrost glass slides (Superfrost Plus, Fisher Scientific, Pittsburgh, PA) facilitated by 4 equidistant radial cuts into the peripheral retina. Wholemounts were immunohistochemically stained immediately at room temperature.

Group 2 rats were euthanized at 21 days by rising concentration of CO_2_ and perfused intracardially with 4% PFA in PBS. Eyes were dissected and immersion fixed in 4% PFA in PBS for 2 h at 4°C and cryoprotected by sequential immersion in 10%, 20% and 30% sucrose solution in PBS, each for 24 h with storage at 4°C. Eyes were orientated to permit radial sectioning and embedded using optimal cutting temperature embedding medium (Thermo Shandon, Runcorn, UK) in peel-away moulds (Agar Scientific, Essex, UK) by rapid freezing under crushed dry ice and stored at −80°C. Eyes were sectioned radially on a cryostat microtome (Bright, Huntingdon, UK) at −22°C at a thickness of 20 µm and sections mounted on positively charged glass slides. Radial eye sections containing the optic disk and thus sectioned at a consistent axis were utilized for subsequent analysis. Sections were stored at −20C°.

### Immunohistochemistry

Wholemounted retinae from Group 1 rats were permeabilized in 0.5% Triton x-100 in PBS for 15 min at −70°C before washing with room temperature 0.5% Triton x-100 for a further 15 min. Retinae were incubated with primary antibodies diluted in wholemount antibody diluting buffer (wADB; 2% bovine serum albumin, 2% Triton x-100 in PBS) overnight at 4°C and, the following day, were washed 3×10 min in PBS and incubated with secondary antibodies in wADB for 2 h at room temperature. After 2 h, retinae were washed for 3×10 min in PBS and mounted with the GCL uppermost on glass slides. Slides were allowed to air dry before mounting in Vectorshield medium (Vector Laboratories, Peterborough, UK) and applying cover slips. The antibodies used in this staining are detailed in Table 2.

Mounted radial retinal sections through the optic nerve head from Group 2 rats were equilibrated to room temperature, hydrated in PBS for 2×5 min, permeabilized in 0.1% Triton x-100 in PBS for 20 min at room temperature and washed for 2×5 min in PBS before encircling with a hydrophobic PAP pen (Immedge pen; Vector Laboratories). Non-specific protein binding sites were blocked by incubating sections in blocking buffer (75 µl; 0.5% bovine serum albumin (g/ml), 0.3% Tween-20, 15% normal goat/donkey serum (Vector Laboratories) in PBS) in a humidified chamber for 30 min at room temperature, drained and incubated with primary antibodies diluted in antibody diluting buffer (ADB; 0.5% bovine serum albumin, 0.3% Tween-20 in PBS) overnight at 4°C. The following day, retinal sections were washed for 3×5 min in PBS and incubated with secondary antibodies diluted in ADB for 1 h in a hydrated incubation chamber at room temperature and then washed for 3×5 min in PBS, mounted in Vectorshield mounting medium containing DAPI (Vector Laboratories) and stored at 4°C before microscopic analysis. The antibodies used in this staining are detailed in Table 2. Omission of primary antibody was used as a staining control.

### Microscopy and analysis

**Figure 1:**
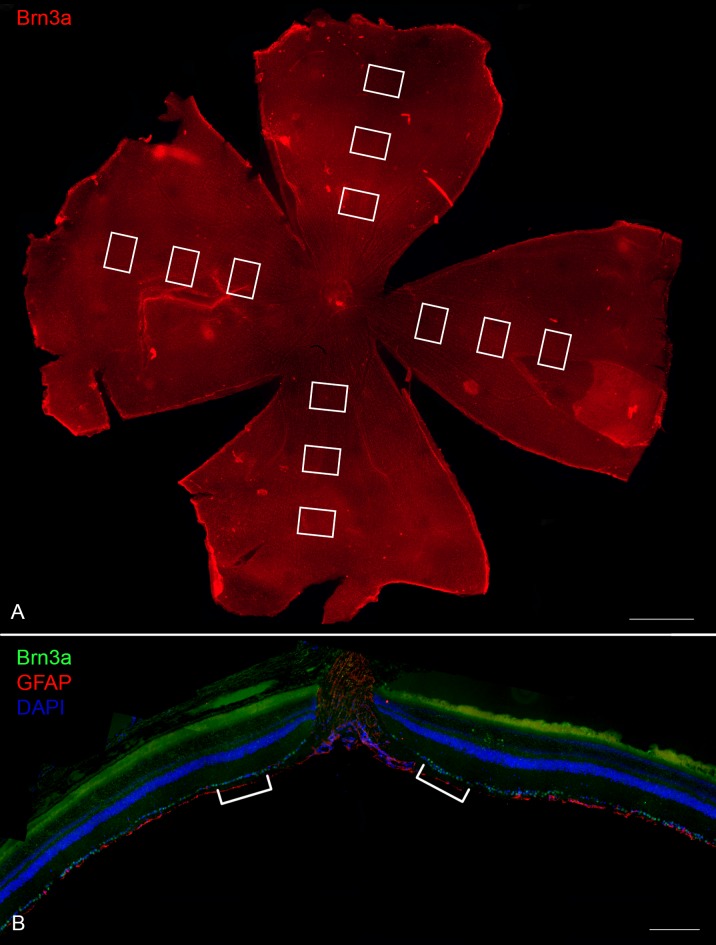
Sampling method for the counting of RGCs in Group 1 wholemounts (A) and Group 2 tissue sections (B). RGC counts in retinal wholemounts were made in sampling boxes (0.260 mm^2^) along quadrant radii at 1.5 mm, 2.5 mm and 3.5 mm from the optic nerve head (white boxes; A). RGCs were counted in sections over 250 µm length regions at a 200–300 µm distance from the centre of the optic nerve in radial retinal sections (white lines; B). For panel A, Brn3a (red) was used to stain the retinal wholemount and RGCs (scale bar: 1 mm). For panel B, Brn3a (*green*) was used to stain RGCs, GFAP (*red*) to stain glia and DAPI (*blue*) was used as a nucleus stain (scale bar: 250 µm).

For Group 1 retinal wholemounts, images were photographed from 3 different regions from each retinal quadrant (as detailed in Fig. 1) using a Zeiss Axioplan-2 fluorescent microscope (Carl Zeiss, Ltd., Hertfordshire, UK). Images were captured at 200X magnification using an Axiocam HRc camera (Carl Zeiss, Ltd.) and all images equivalently contrast enhanced using Photoshop CS3 (Adobe Systems, Inc., San Jose, CA). The experimenter was blinded to treatment group and retinal region during counting of total numbers of Brn3a^+^ and FG^+^ cells (total = 3 counts/quadrant, 12 counts/retina and 6 retinae from 6 different animals per treatment group) observed in each 0.260 mm^2^ imaged area. The mean number of RGCs/image was calculated from a mean count at each of the 3 radial distances as well as a mean composite count over all 3 distances.

For Group 2 retinal sections, images were photographed as detailed above and analysed by an operator blinded to treatment groups. Brn3a^+^, βIII-tubulin^+^ and Islet-1^+^ RGCs were counted in 20 µm-thick radial sections of the retina, along a 250 µm linear region of the GCL, either side of the optic nerve head (Fig. 1). Four sections/retina and 6 retinae from 6 different animals per treatment group were quantified. As sectioned tissue inevitably included split RGC nuclei, these were only included in the counts if the sizes of Brn3a^+^//Islet-1^+^ stained nuclei were similar to the co-localised DAPI^+^ nuclei. Changes in soma/nuclear size have not been reported after ONC [32], therefore, although this technique would be ineffective at calculating absolute RGC numbers, any inaccuracies would apply equally to both conditions and thus would not impede reliable comparisons of RGC loss between groups.

### Statistics

All statistical tests were performed using SPSS 17.0 (IBMM SPSS, Inc., Chicago, IL) and data were presented as mean ± standard error of the mean (SEM). The Shapiro-Wilk test was used to ensure all data were normally distributed before applying a one-way analysis of variance (ANOVA) with a Tukey *post-hoc* test. Statistical differences were considered significant at p values <0.05.

At the end of the study, a power calculation was performed to determine the required optimal animal numbers per treatment group for animal Group 1 and 2. Power calculations were performed using G*Power [33] with the following parameters: α error probability (P value) of 0.05, power (1 - β error probability) of 0.95 and effect size of 1.32, 2.77 and 7.64 which relates to 10%, 20% and 50% RGC death (as determined by the program) respectively compared to intact counts of 17.83 RGC/250 µm of retina.

## Results

### Group 1 rats

#### FG^+^/Brn3a^+^ RGCs in retinal wholemounts

**Figure 2:**
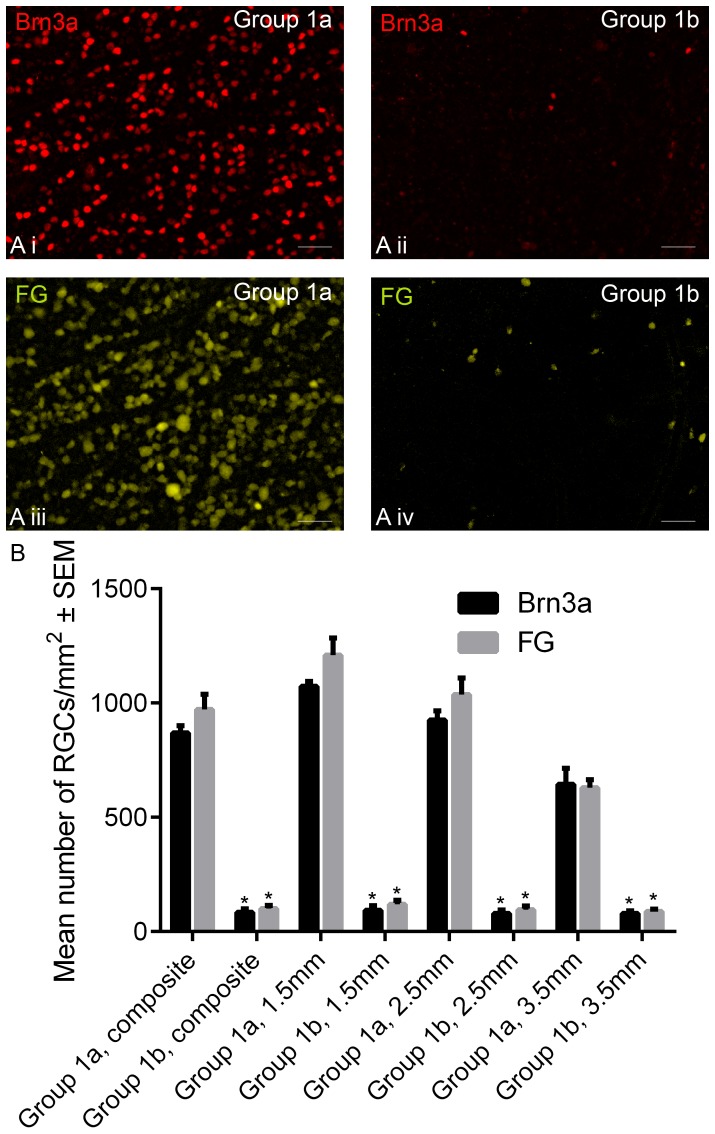
Brn3a^+^ and FG^+^ RGC counts in Group 1 wholemounted retinae. Immunohistochemically stained wholemounted retina, stained for Brn3a (*red*; **Ai** and **Aii**) and FG (*gold*; **Aiii** and **Aiv**; photographs taken in the same field, respectively), taken from Group 1a animals (intact optic nerve; **Ai** and **Aiii**) and Group 1b animals (21 days after ONC; **Aii** and **Aiv**). All images are representative of the 12 images taken per retina from 6 different animals (*scale bar*: 50 µm). In (**B**), the mean number of Brn3a^+^ and FG^+^ RGCs in a 1 mm^2^ region, from Group 1a and Group 1b rats, calculated as an composite average of 12 images, or an average of 4 images taken at 1.5 mm, 2.5 mm and 3.5 mm from the optic nerve head. *Asterisks* indicate significant difference at p<0.01 between the ONC counts and their respective Group1a controls.

In the retinae of Group 1a eyes (intact optic nerve), mean numbers/mm^2^ of Brn3a^+^ and FG^+^ RGCs were 869.7±30.6 and 971.7±67.0, respectively (Fig. 2). In the retinae of Group 1b eyes (21 days after ONC), mean numbers/mm^2^ of Brn3a^+^ and FG^+^ RGCs were significantly reduced (P<0.05) compared to Group 1a values (83.8±15.0 and 100.9±13.8, respectively). In Group1a retinae, the density of RGCs decreased towards the periphery, reflected by a mean number/mm^2^ of 1073.1±21.9, 926.1±39.5 and 644.7±69.1 Brn3a^+^ RGCs and 1209.9±75.0, 1036.4±73.3 and 629.0±35.3 FG^+^ RGCs at 1.5 mm, 2.5 mm and 3.5 mm from the centre of the optic nerve, respectively. RGC number was significantly less (P<0.05) in Group 1b retinae compared to Group 1a retinae with mean counts of 92.9±19.9, 79.3±15.5, 79.2±11.3 Brn3a^+^ RGCs and 119.1±17.3, 95.8±15.6, 88.1±10.3 FG^+^ RGCs at 1.5 mm, 2.5 mm and 3.5 mm from the centre of the retina, respectively.

#### FG/Brn3a double staining in retinal wholemounts of Group 1a

In the retinae of Group 1a eyes (intact optic nerve), quantification of RGCs double staining for Brn3a and FG revealed that 2.0±0.15% of Brn3a^+^ RGCs were FG^-^ and 12.1±0.7% of FG^+^ RGCs were Brn3a^-^. These data confirm that Brn3a is a specific marker of RGCs.

### Group 2 rats

#### Brn3a^+^, βIII-tubulin^+^ and Islet-1^+^ RGCs in radial retinal sections

The somata, dendrites and axons of RGCs were stained with βIII-tubulin in the GCL, inner plexiform layer and nerve fibre layer, respectively, and Islet-1-stained nuclei were present in the GCL as well as in the INL, whilst Brn3a exclusively stained RGC nuclei in the GCL.

**Figure 3:**
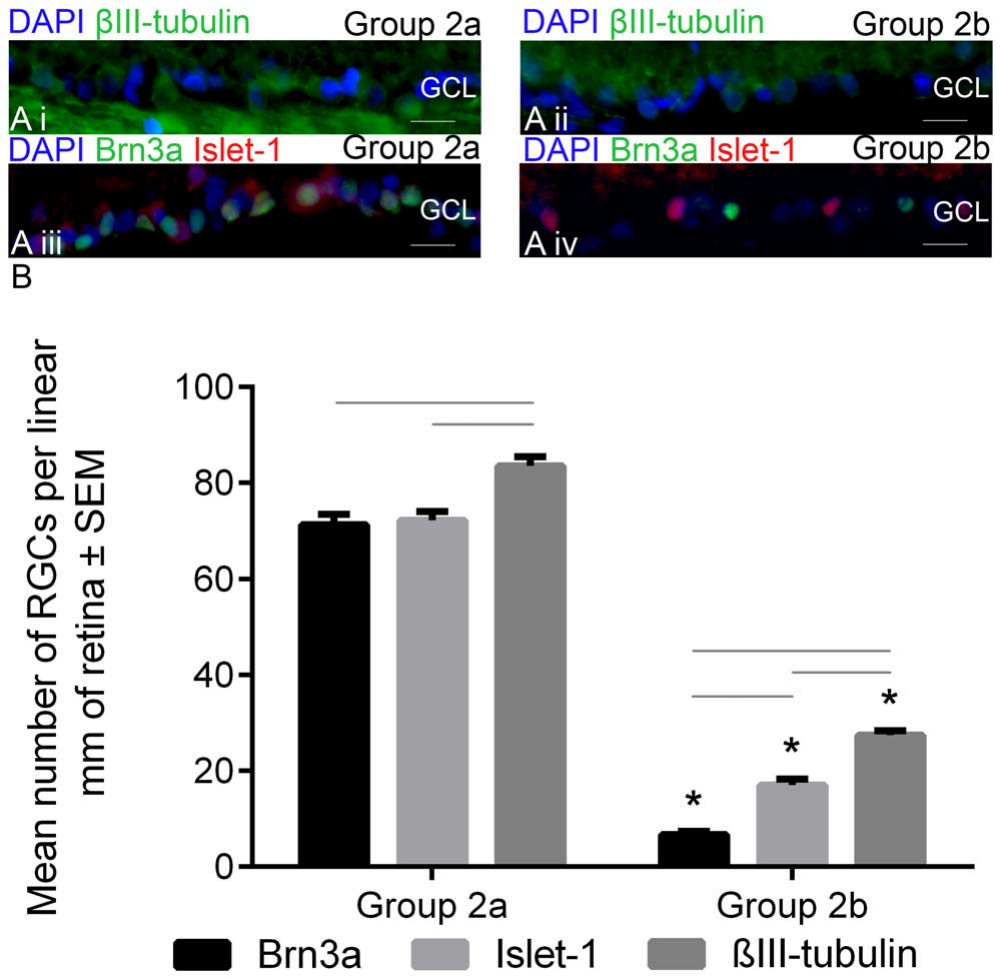
Brn3a^+^, Islet-1^+^ and βIII-tubulin^+^ RGC counts in Group 2 radial retinal sections from Group 2a eyes (intact optic nerves) and Group 2b eyes (21 days after ONC). Immunohistochemically stained 20-µm-thick radial sections of retina through the optic nerve head, stained for βIII-tubulin (*green*; **Ai and Aii**) or Brn3a (*green*) and Islet-1 (*red;* **Aiii and Aiv**), taken from Group 2a eyes (**Ai** and **Aiii**) and Group 2b eyes (**Aii** and **Aiv**) with the ganglion cell layer (GCL) labelled. All images are representative of the two images/section, four sections/retina, and six retinae prepared from six different animals/group. DAPI was used as a nucleus stain (*scale bar*: 15 µm). In (**B**), the number of Brn3a^+^, Islet-1^+^ and βIII-tubulin^+^ cells, in a 1 mm linear region of the GCL from Group 2a/2b eyes. *Asterisks* indicate significant difference at p<0.05 between groups (ONC and intact) and *black lines* indicate significant difference at p<0.05 within groups.

In Group 2a retinae (intact optic nerve), the numbers/mm of Brn3a^+^, Islet-1^+^ and βIII-tubulin^+^ RGCs in the GCL were 71.3±2.2, 72.3±1.8 and 83.6±1.9, respectively (Fig. 3). In Group 2b retinae (21 days after ONC), the numbers/mm of Brn3a^+^, Islet-1^+^ and βIII-tubulin^+^ RGCs in the GCL were 6.7±0.7, 17.0±1.3 and 27.4±0.9, respectively; which were all significantly different (P<0.001) from the respective Group 2a counts (Fig. 3).

The number of βIII-tubulin^+^ RGCs counted in the GCL was significantly reduced (P<0.001) in Group 2b retinae when compared to Group 2a retinae, but remained significantly higher (P<0.05) than the number of either Islet-1^+^ or Brn3a^+^ RGCs in the GCL of Group 2b retinae. Similarly, the number of Islet-1^+^ RGCs in the GCL was significantly reduced 21 days after ONC, but the number was significantly higher (P<0.05) than the number of Brn3a^+^ RGCs counted in the GCL after ONC. These data demonstrate that βIII-tubulin and Islet-1 overestimate RGC numbers.

#### Co-localization of Brn3a^+^, βIII-tubulin^+^ and Islet-1^+^ RGCs with GFAP, Syntaxin-1 and ED1 in radial retinal sections

**Figure 4:**
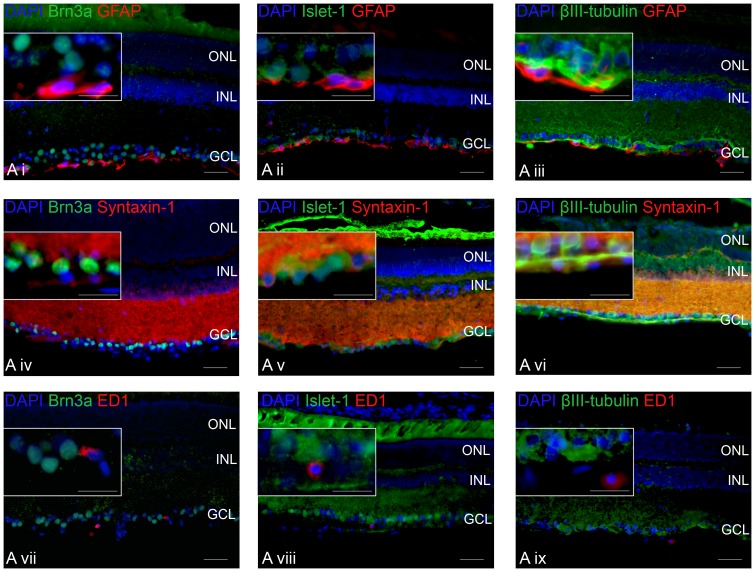
Expression of Brn3a, Islet-1 and βIII-tubulin in radial retinal sections and co-localisation of staining for the astrocyte marker GFAP, the amacrine cell marker Syntaxin-1 and the macrophage/microglia marker ED1. Immunohistochemically stained 20-µm-thick radial sections of retina, stained for Brn3a (*green;* ***Ai, Aiv and Avii***), Islet-1 (*green*; **Aii, Av and Aviii**), βIII-tubulin (*green*; **Aiii, Avi and Aix**), GFAP (*red*; **Ai, Aii and Aiii**), Syntaxin-1 (*red*; **Aiv, Av and Avi**) and ED1 (*red*; **Avii, Aviii and Aix**), taken from Group 2a eyes (intact optic nerve), with the outer nuclear layer (ONL), inner nuclear layer (INL) and ganglion cell layer (GCL) labelled, insert showing higher power image of the GCL. All images are representative; DAPI (*blue*) was used as a nucleus stain (*scale bar*: 50 µm; *inset scale bar*: 25 µm).

GFAP^+^ astrocytes were a distinct population of cells in the GCL that did not stain with Brn3a, Islet-1 or βIII-tubulin. Syntaxin-1^+^ amacrine cells in the INL and GCL were Brn3a^-^, although some were Islet-1^+^ and βIII-tubulin^+^ (Fig. 4). ED1^+^ stained sparsely distributed macrophages/microglia throughout Group 2a retinae, which did not stain with Brn3a-, Islet-1- or βIII-tubulin. These data suggest that the overestimation of RGC numbers by βIII-tubulin and Islet-1 staining is due to the staining and subsequent counting of amacrine cells displaced into the GCL.

### Group 1 and 2 rats

#### Estimates of RGC death were similar in Group 1 Brn3a/FG-stained retinal wholemounts and Group 2 Brn3a-stained radial sections

**Figure 5:**
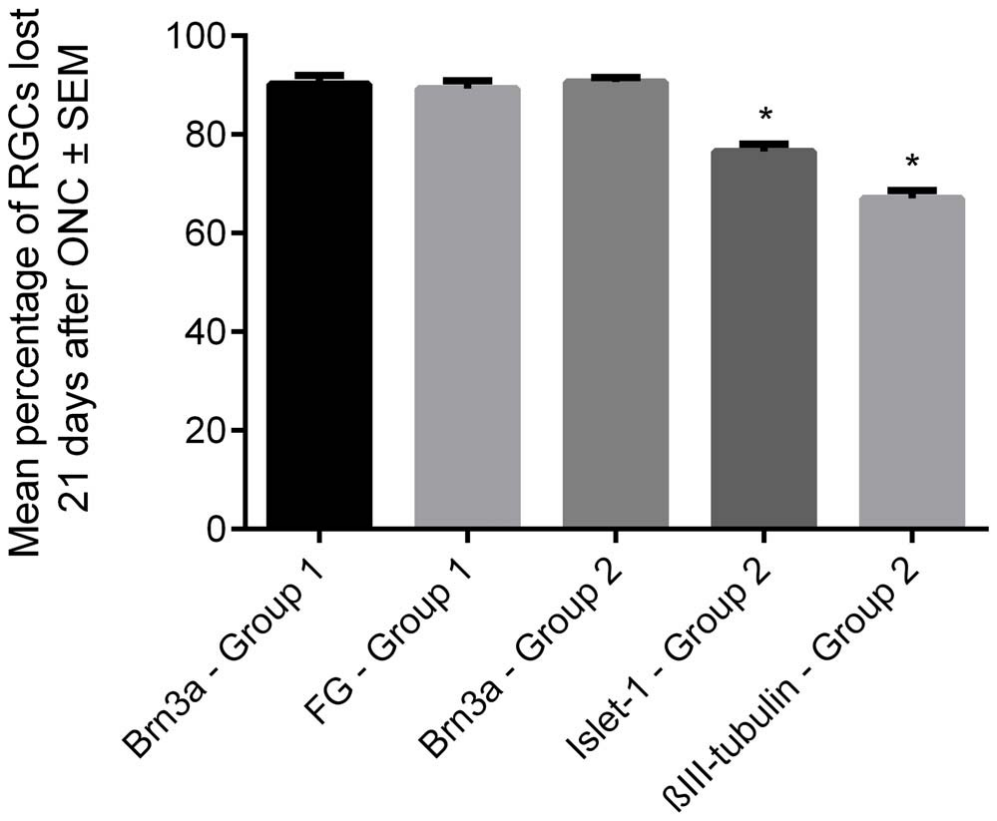
The mean percentage of RGC loss 21 days after ONC in Group 1 and Group 2 rats. *Asterisks* indicate significant difference at P<0.05 compared to Brn3a^+^ RGCs in sections and Brn3a^+^/FG^+^ RGCs in wholemounts (n = 6).

In Group 2 radial retinal sections, the percentage death of Islet-1^+^/βIII-tubulin^+^ RGC detected was 76.5±1.5% and 67.0±1.6%, respectively, which was significantly lower (P<0.05) than that revealed by Brn3a staining (90.6±0.9%) as well as that estimated after Brn3a/FG staining of retinal wholemounts (90.2±1.8% and 89.3±1.6%, respectively; Fig. 5). These data show that Brn3a-stained radial sections report the same percentage RGC death as Brn3a-/FG-stained wholemounts demonstrating that both techniques reliably quantify RGC loss. In contrast, Islet-1-/βIII-tubulin-stained radial sections overestimate the number of surviving RGCs after ONC and thus underestimate the percentage of RGC loss.

#### Brn3a-stained Group 1 wholemounts and Group 2 radial sections detect as low as 10% RGC death

Using data derived from this experiment, a power calculation was performed to determine that, in order to detect 50% RGC death at P<0.05, 3 animals/group are required, to detect 20% RGC death at P<0.05, 5 animals/group are required and to detect 10% RGC death at P<0.05, 16 animals/group are required. These data suggest that even as low as 20% RGC death can be detected in radial stained sections with acceptable animal numbers.

## Discussion

Reliable measures of percentage RGC survival in experimental degenerative retinal conditions including glaucoma [34] and optic nerve neuropathies [35] are essential to evaluate the efficacy of novel therapeutic agents designed to treat these disorders. Pertinent to this is the identification of good phenotypic markers for RGCs. This study provides evidence that, in determining percentage RGC loss in rats, estimates from radial sections of retinae are similar to estimates from wholemounted retinae. Furthermore, the data reinforce the notion that Brn3a antibodies are the most reliable phenotypic markers of RGCs both in wholemounts and sections, whereas βIII-tubulin and Islet-1 antibodies stain amacrine cells in the GCL, giving underestimates of RGC loss.

Our findings that Brn3a antibodies and FG tracer label statistically similar numbers of RGCs in wholemounted retinae, both before and after ONC-induced RGC death are consistent with the reported 95% co-localization of Brn3a and FG staining in RGCs [18], further emphasising the utility of Brn3a antibodies as a reliable RGC marker. The percentage of Brn3a^+^ cells that were FG^-^ was 2.0%, which is comparable to the 4.4% previously reported [18]. FG, a weak base, freely diffuses into intracellular vesicles where it raises the pH, making the vesicle impermeable, trapping the FG within; the vesicle is then transported along the cytoskeleton of the axon [36]. A likely explanation for the presence of FG^-^Brn3a^+^ RGCs is that FG uptake into RGC axons is 98% efficient. In this study the percentage of FG^+^ cells that were Brn3a^-^ was 12.1%, slightly more than the 3.4% previously reported [18], a figure that is probably explained by the exclusion of FG^+^/Brn3a^-^ macrophages/microglia from the data in the Nadal-Nicolas study. By contrast, in the present study, all cells that were positive for their respective phenotypic markers were counted and included and thus we can postulate (but not be certain) that a small proportion of these FG^+^ cells were macrophages/microglia. In Figure 2, FG^+^ and Brn3a^+^ images were taken in the same field but co-localization appears reduced after ONC, likely due to surviving FG^+^/Brn3a^-^ macrophages the frequency of which is increased after RGC death. Although RGC number decreased with increasing distance from the optic nerve head (1.5 mm, 2.5 mm and 3.5 mm), the number of RGCs surviving after ONC did not change with distance (Fig. 2B.), and thus counts closest to the optic nerve head yielded the biggest relative differences. This is explained by the homogenous RGC death occurring after axotomy/ONC [10] whereas the sectorial death seen in laser-induced ocular hypertension models of glaucoma [37]–[40] requires sampling from multiple distances to ensure accuracy in determining the extent of RGC loss. The counts made from 4 and 12 images had similar SEM, revealing the strength of the sampling method.

One limitation of this study is that eyes with intact optic nerves (Group 1a, 2a) contralateral to the ONC eyes (Group 1b, 2b) were used as controls. It has previously been shown that ocular hypertension induces microglia activation in eyes contralateral to the injury [41] as well as a decrease in astrocytes and activation of Müller cells [42], with these results partially replicated for unilateral ONC [43]. However, these changes have not been correlated with RGC number [42] and equally, the RGC loss observed in our study 21 days after ONC (when compared to contralateral eyes) is equivalent to losses reported in the published literature [3], [35], [44] leading us to assume that any contralateral effects instigated by the unilateral ONC had negligible effects on RGC numbers. An added value of contralateral eye counts is the resulting reduction in animal usage.

In wholemounted Group 1 retinae, Brn3a is preferred to FG as a RGC marker, since FG delivery into the optic nerve for retrograde transport to RGC somata requires further surgery under anaesthesia. Counts of RGCs with 4 sampling areas each 1.5 mm from the optic nerve head gave reliable estimates of RGC number in which SEM values where similar to when 12 images/samples were used. In wholemounts, βIII-tubulin-stained axons and dendrites which obscured many RGCs, leading to their exclusion from the count (Fig. S1). In Islet-1-stained wholemounts, the high background staining of the INL (Fig. S1) made RGC counting difficult and excluded consistent 4-image sampling of some retinae.

Retinal wholemounts restricts the use of the retina to a limited number of antibody stains [45] and renders the tissue unusable for further analysis, such as evaluating the effects of treatments on other proteins and cells as well as retrieving morphological data such as retinal detachment or the location of grafted cells. By contrast, radial sections allow all of these data to be obtained from multiple sections from the same eyes with no loss of fidelity in RGC quantification. Of note, the homogenous and diffuse loss of RGCs seen after ONC/axotomy [10] makes sampling around the optic nerve head in sections a reliable estimate of RGC loss, whereas in models such as glaucoma where the loss is sectorial [37]–[40], expanded sampling is required to include sections throughout the eye, counting in regions at multiple distances from the optic nerve head.

We tested Brn3a [18], [24], Islet-1 and βIII-tubulin [7], [9], [24] as presumptive phenotypic RGC markers. Compared to Brn3a, βIII-tubulin-stained significantly more cells in the GCL both before and after ONC-induced RGC death, which we and others [8] interpret as being due to labelling of amacrine cells in addition to RGCs. Thus this antibody over estimates the numbers of RGCs both in Group 1a/2a animals (intact optic nerve) and even more so in Group 1b/2b animals (21 days after ONC) where the ratio of amacrine cells to RGCs is increased as RGCs are preferentially lost [20]. Compared to the Brn3a antibody, the Islet-1-stained similar numbers of cells before ONC but significantly more after ONC (although still significantly less than with βIII-tubulin staining after ONC). Since the Islet-1 antibody stains RGCs and amacrine cells [9], and more displaced amacrine cells survive relative to RGCs in the GCL 21 days after ONC [20], Islet-1 antibody will overestimate RGC numbers in comparison to Brn3a after ONC-induced RGC death.

To determine if counts from sections are as reliable as from wholemounts in quantifying RGC death, we compared the percentage loss of RGCs between all methods used in this study. The published figures of 90% RGC death 21 days after ONC [24], [35], [44] are similar to those seen in this study with Brn3a-/FG-stained wholemounts and with Brn3a-stained radial retinal sections, confirming that counts made in retinal sections are as reliable as those made in retinal wholemounts. Considering both the small SEM for each mean and the results from our power calculation, counts from sections and wholemounts have equal fidelity in determining significant differences between groups with as low as 20% difference in RGC number, without the need for significant increases in animal numbers per treatment group.

## Conclusions

We conclude that Brn3a antibody staining of radial retinal sections is a preferred method for estimating RGC frequency when compared to techniques using retinal wholemounts in rodent models of ocular disease. The method avoids both the additional surgery needed for injection of FG and the sacrifice of further molecular and morphological retinal analysis, potentially reducing animal usage and associated costs. We show that cell counts from retinal sections are as reliable as those from retinal wholemounts and have equivalent fidelity in determining RGC loss between control and experimental groups.

## Supporting Information

Figure S1 **Islet-1- and βIII-tubulin-stained wholemounted retinae from Group 1a eyes (intact optic nerve).** Immunohistochemically stained wholemounted retina stained for Islet-1 (*green*; **Ai**) or βIII-tubulin (*green*; **Aii**), taken from Group 1a eyes. All images are representative of the 12 images taken per retina from 6 different animals (*scale bar*: 50 µm).
(TIF)
