# Correction: Comparative Evaluation of Methods for Estimating Retinal Ganglion Cell Loss in Retinal Sections and Wholemounts

**DOI:** 10.1371/journal.pone.0116116

**Published:** 2014-12-19

**Authors:** The PLOS ONE Staff

## Notice of Republication

This article was republished on November 20th, 2014, due to the Figure 1 being corrupted in the online version of this article. The publisher apologizes for this error.
