# Supplementary figures and images for: Comparative Evaluation of Methods for Estimating Retinal Ganglion Cell Loss in Retinal Sections and Wholemounts
Source: PLoS One. 2014 Oct 24;9(10):e110612. doi: 10.1371/journal.pone.0110612 (PMC4208790; doi:10.1371/journal.pone.0110612)

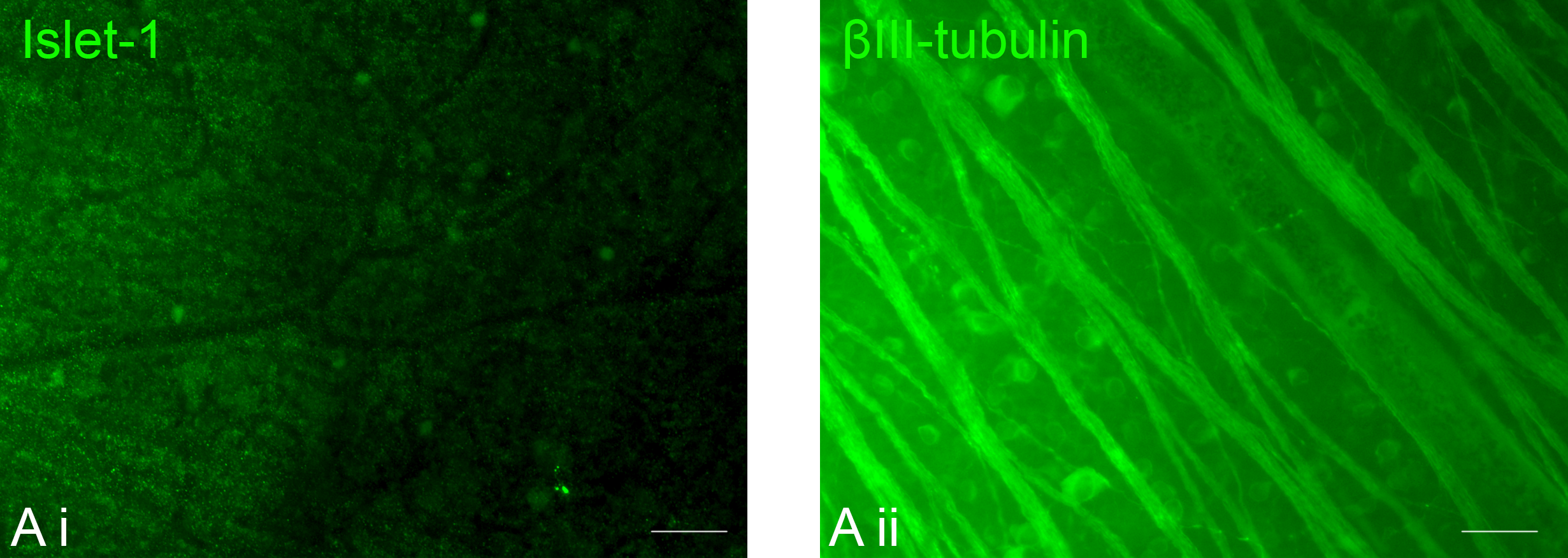

Supplement: Figure S1 — Islet-1- and βIII-tubulin-stained wholemounted retinae from Group 1a eyes (intact optic nerve). Immunohistochemically stained wholemounted retina stained for Islet-1 (green; Ai) or βIII-tubulin (green; Aii), taken from Group 1a eyes. All images are representative of the 12 images taken per retina from 6 different animals (scale bar: 50 µm). (TIF) [file pone.0110612.s001.tif]
